# Employment sustainability after return to work among Japanese stroke survivors

**DOI:** 10.1007/s00420-018-1319-2

**Published:** 2018-05-25

**Authors:** Motoki Endo, Yasuo Haruyama, Go Muto, Kazuhito Yokoyama, Noriko Kojimahara, Naohito Yamaguchi

**Affiliations:** 1 Department of Public Health, Tokyo Women’s Medical University, Tokyo, 162-8666 Japan; 2 Department of Public Health, Dokkyo Medical University, Mibu, Japan; 3 Department of Epidemiology and Environmental Health, Juntendo University Faculty of Medicine, Tokyo, Japan

**Keywords:** Return to work (RTW), Stroke survivors, Work continuance rate, Recurrent sickness absence (RSA), Resignation

## Abstract

**Purpose:**

Few studies have investigated the work continuance rate among stroke survivors who return to work (RTW). The objective of this study was to investigate work sustainability after RTW and the causes of recurrent sickness absence (RSA) among Japanese stroke survivors.

**Methods:**

Data on stroke survivors were collected from an occupational health register. The inclusion criteria were as follows: employees who were aged 15–60 years old and returned to work after an episode of sick leave due to a clinically certified stroke that was diagnosed during the period from 1 January 2000 through 31 December 2011.

**Results:**

284 employees returned to work after their first episode of stroke-induced sick leave. The work continuance rate for all subjects was 78.8 and 59.0% at one and 5 years after the subjects’ RTW, respectively. After returning to work, the subjects worked for a mean of 7.0 years. Of 284 employees who returned to work, 86 (30.3%) experienced RSA. The RSA were caused by recurrent strokes in 57.0% (49/86) of cases, mental disorders in 20.9% (18/86) of cases, and fractures (often due to accidents involving steps at train stations or the subject’s home) in 10.5% (9/86) of cases. 21 employees resigned after returning to work. The resignation rates at 1 and 5 years were 4.9 and 7.6%, respectively. According to the multivariate analysis including all variables, the subjects in the ≥ 50 year group were at greater risk of work discontinuation than the ≤ 49 year (reference) age group (HR: 2.26, 95% CI 1.39–3.68).

**Conclusions:**

Occupational health professionals need to provide better RTW support to stroke survivors and should pay particularly close attention to preventing recurrent strokes, mental disorders, and fractures.

## Introduction

While the incidence and mortality rates of stroke have been declining over time due to improvements in the treatment of the condition, stroke remains the fourth leading cause of death in Japan (Ministry of Health 2011). Stroke is still a serious condition as it causes death, various disabilities, and substantial reductions in quality of life in both developed and developing countries (Endo et al. 2016; Gilworth et al. 2009). Stroke survivors can experience severe brain function impairments, such as hemiplegia, aphasia, dysphagia and anarthria.

In developed countries, about 20% of strokes occur in people of working age (20 to 65 years old), many of whom are in paid employment (Arauz 2013; Luengo-Fernandez et al. 2009). Due to ageing populations and the prolonged survival of stroke patients, the prevalence of stroke survivors within the working-age population is expected to increase in the future (Ministry of Health Labor 2016).

For young stroke survivors, employment is one of the most important factors affecting their economic, mental, and social well-being (Arauz 2013). The ability to return to work (RTW) after a stroke is an important issue for stroke survivors within the working-age population (Morris 2011). While returning to work is generally considered to be closely related to a complete recovery, the situation is more complicated than that because symptom severity is roughly associated with patterns of sickness absence (SA) and returning to work (Roelen et al. 2010). In Japan, after the Ministry of Health, Labor, and Welfare issued guidelines relating to RTW support in February 2016, it seems that there has been more interest in following-up stroke survivors after their RTW (Ministry of Health Labor 2016).

However, few workforce-based studies have investigated the work continuance rate after RTW among stroke survivors. The objective of this study was to clarify the work sustainability of Japanese stroke survivors after RTW and to investigate the most common causes of recurrent sickness absence (RSA) and to analyze the predictors of the time to RSA or resignation after stroke survivors RTW.

## Methods

### Study design and data regarding SA in this study

This was a workforce-based cohort study of the work continuance rate of stroke survivors after (initial) stroke-induced SA in Japan. The subjects of this study were employees of large-scale companies who had suffered strokes. We collected anonymized data from a private occupational health center, as described in our previous study (Endo et al. 2015, 2016). The occupational health center contracted occupational physicians (OP) to provide employees who belonged to a large Japanese corporate group, which included various companies (e.g., telecommunications, logistics, energy, and construction companies). About 68,000 employees worked for these companies on a full-time basis from 2000 to 2011.

Employees who needed to be absent due to any medical condition submitted physicians’ certificates to the human resources department (HR); for example, they submitted a certificate stating ‘this employee cannot work due to stroke’. The HR sent a copy of each certificate to the occupational health center.

The OP at the occupational health center confirmed the medical validity of the physician-issued certificate, and the certificate for SA was returned to the HR department. The OP recorded the cause of each SA based on the World Health Organization’s 10th International Classification of Diseases (ICD-10).

As for the RTW data, employees who would like to RTW after being treated for a stroke were required to submit a physician’s certificate to the HR, for example, a certificate saying ‘this person can return to work after 5 February, although a gradual RTW is desirable’. The HR sent a copy of the certificate to an OP for further confirmation that the employee’s RTW was medically acceptable, and to arrange a RTW interview involving the employee, their superior, the OP, and the HR. The employee’s company then determined whether the employee could RTW, whether they would work full-time or part-time, and what kinds of task they could perform. When making RTW decisions, the employee’s company referred to the physician’s certificate, the OP’s certificate, and the company’s needs. If a RTW was permitted, an OP recorded the day of the employee’s RTW, the disease they were suffering from, whether they were a full-time or part-time employee, etc., in the Health Register System.

After an employee’s RTW, an OP interviewed them 1–2 months later to check their physical and mental condition. After this interview, the OP submitted an OP certificate to the HR, which stated something like “from 1 March, this person can work on a full-time basis”. RSA after RTW were only allowed after a physician’s certificate stating that ‘this person cannot work’ had been obtained.

Therefore, the data collected for this study were based on physicians’ certificates rather than self-reported data.

### Subjects and the inclusion criteria

The inclusion criteria for this study were as follows.

Employees that were registered in the Health Data System, aged 15–60 years, and returned to work after their first SA due to a stroke (stroke included “cerebral infarction (I63)”, “cerebral hemorrhage (I61)”, and “subarachnoid hemorrhage (I60)”; ICD-10, based on a physician’s certificate) that occurred between January 1, 2000, and December 31, 2011.

The subjects of this study were employees who returned to work after a SA due to a clinically certified stroke that was diagnosed between 1 January 2000 and 31 December 2011. During this 12-year period, 382 employees had strokes. In each case, the employee’s first SA was not due to a recurrent stroke because employees who had previously experienced SA due to strokes that occurred before 31 December 1999 were not included in this study.

### The start day and censoring criteria for the survival analysis

The first day of the subject’s RTW after their stroke-induced SA was used as the start of the period examined during the survival analysis.

The survival analysis was censored at either the end of the follow-up period (December 31, 2012) or the day of the employee’s retirement (March 31 of the year that they became 60 years old), whichever came first. In this analysis, event days were defined as the first day of RSA due to any physician-certified illness or the day on which the subject resigned before their retirement (60 years old). In cases in which the subject died, the day of their death was regarded as a day of RSA.

### Statistical analysis

The work continuance rate was defined as the frequency of sustained work, without RSA or resignation, after RTW and was analyzed using Kaplan–Meier survival analysis. The number of person-days was calculated based on the follow-up period. The work continuance rate was measured at 1–5 years after the employees’ RTW using Kaplan–Meier analysis. Since RSA and resignation act as competing factors in post-RTW survival analyses, a survival analysis with competing risks was performed using EZR, which is statistical analysis software provided by CRAN (The Comprehensive R Archive Network) (Kanda 2013).

We employed a Cox regression model to analyze the risk factors for work discontinuation based on hazard ratios (HR) and their 95% confidence intervals (95% CI). A HR of more than 1 indicated a shorter time to work discontinuation (RSA or resignation), compared with the reference. A HR of less than 1 indicated a longer time to work disability (RSA or resignation), compared with the reference.

Age, sex, the location of the company, whether the employee was a desk worker or manual worker, whether the employee was a manager, stroke subtype, and the duration of SA were examined as potential risk factors for work discontinuation. The subjects were stratified into two age groups: ≤49 years (reference) and ≥ 50 years. A ‘manager’ was defined as an individual in an administrative post, which in Japanese organizations is considered to be a higher position than a section chief. Job titles were divided into two groups: ‘desk workers’ (for example, ‘office workers’, ‘sales workers’, ‘researchers’), who performed jobs that mainly involved a mental workload, and ‘manual workers’ (for example, ‘technicians’), who performed jobs that mainly involved a physical workload. The subjects were stratified into three SA duration groups: ≤ 60 days (reference), 61–120 days, and ≥ 121 days.

We performed univariate and multivariate analyses including all of the abovementioned variables using IBM SPSS for Windows Ver. 24.

### Ethics

This study was approved by the medical ethics committee of Tokyo Women’s Medical University (number: 3244).

## Results

In total, 359 employees experienced their first episode of SA due to a physician-certified stroke during the study period. Of these employees, 284 returned to work after their first stroke-induced SA. Table 1 shows the subjects’ basic characteristics: 251 (88.3%) of 284 were male, and 33 (11.7%) were female, and their mean age on the first day of their SA was 52.0 years. The mean duration of the initial SA was 134.5 days (approximately 4.5 months).

**Table 1:** Basic characteristics of the stroke survivors in this study

	Total *n* (%)	Mean age (SD) (years)	Mean duration of the first SA (days)	Experienced RSA (*n* = 86)	RSA due to stroke (AMI)	RSA due to mental disorders	RSA due to fractures	Resigned (*n* = 21)	Median duration of work after RTW (years)
Age									
≤ 49	80	44.6 (5.4)	132.1	23	11 (1)	6	4	1	–
≥ 50	204	54.8 (2.9)	135.4	63	38 (1)	12	5	20	5.1
Sex									
Male	251	51.9	131.8	80	44 (0)	17	9	15	7.1
Female	33	52.8	155.7	6	5 (2)	1	0	6	4.4
Company location									
Rural area	83 (29.2)	53.1 (4.2)	129.4	25	15 (1)	6	3	6	7.2
Urban area	201 (70.7)	51.5 (6.5)	136.6	61	34 (1)	12	6	15	7.0
Desk worker/manual worker									
Desk worker	212 (74.6)	51.8 (6.3)	144.7	61	34 (2)	10	7	17	6.9
Manual worker	72 (25.3)	52.6 (4.6)	104.5	25	15 (0)	8	2	4	7.1
Manager/non-manager									
Non-manager	264 (93.0)	52.1 (6.0)	137.5	82	47 (2)	17	8	20	7.0
Manager	20 (7.0)	50.2 (5.2)	94.2	4	2 (0)	1	1	1	–
Stroke subtype									
Cerebral infarction	160 (56.3)	52.1 (5.5)	100.9	49	28 (1)	12	2	12	7.0
Cerebral hemorrhage	75 (26.4)	52.2 (6.9)	199.3	24	13 (1)	3	5	8	5.1
Subarachnoid hemorrhage	49 (17.3)	51.3 (5.8)	145.0	13	8 (0)	3	2	1	–
Duration of SA									
≤ 60 days	124 (43.7)	52.3 (5.7)		40	25 (1)	8	1	6	–
> 60 and ≤ 120 days	69 (24.3)	51.1 (7.5)		21	12 (1)	4	3	2	9.8
> 120 days	91 (32.0)	52.2 (4.7)		25	12 (0)	6	5	13	5.3
All strokes	284	52.0 (5.9)	134.5	86	49 (2)	18	9	21	7.0

### Work continuance rates and predictors of work discontinuation after RTW among stroke survivors

**Fig. 1:**
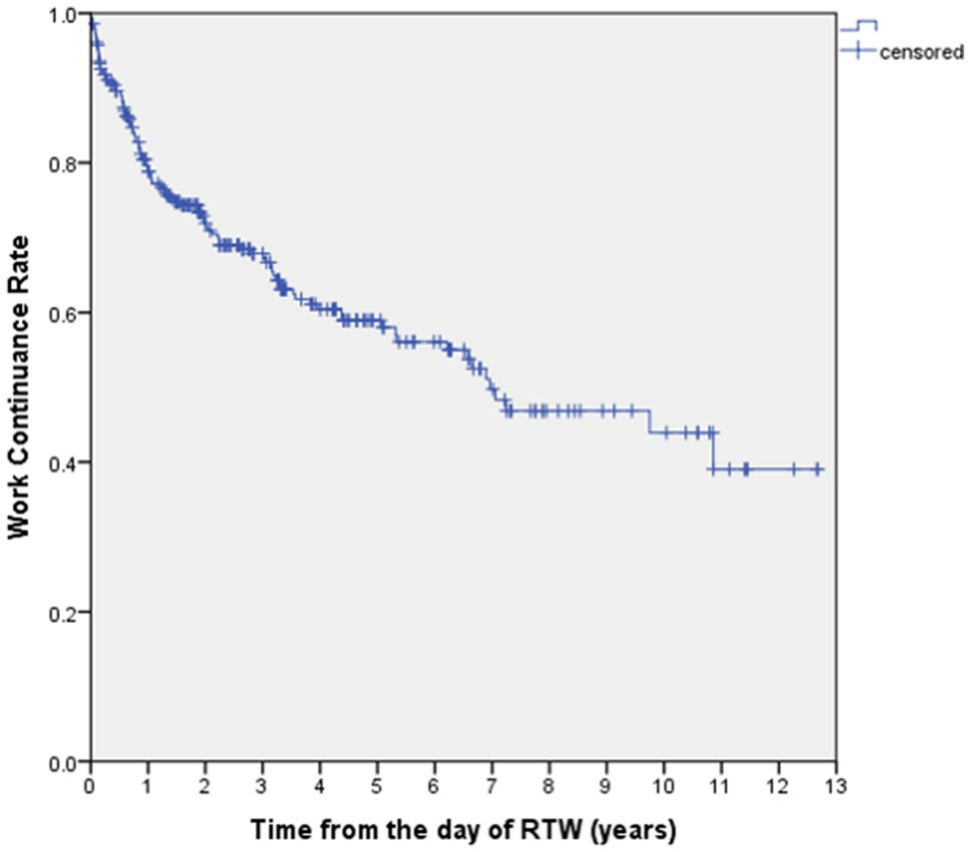
Work Continuance Rate after RTW among Japanese stroke survivors

The Kaplan–Meier survival curve for the work continuance rate after RTW is shown in Fig. 1.

The work continuance rates at 1–5 years were 78.8, 71.9, 67.8, 60.5, and 59.0%, respectively. As shown in Fig. 1, there was a steep reduction in the work continuance rate the first year after RTW.

Table 2 shows the predictors of the time to RSA or resignation by age, sex, company location, desk worker/manual worker, manager/non-manager, stroke subtype, and the duration of SA. None of the examined factors, except for age, significantly affected the time to RSA or resignation. According to the multivariate analysis including all variables, the subjects in the ≥ 50 year group were at greater risk of work discontinuation than the ≤ 49 year (reference) age group (HR: 2.26, 95% CI 1.39–3.68).

**Table 2:** Univariate and multivariate analyses of predictors of work discontinuation among Japanese stroke survivors

Variables	Categories	Univariate analysis		Multivariate analysis	
		HR (95% CI)	*p* value	HR (95% CI)	*p* value
Age					
	≤ 49 (ref)	1		1	
	≥ 50	2.16 (1.34–3.47)	0.001	2.26 (1.39–3.68)	0.001
Sex					
	Male	1		1	
	Female	1.13 (0.62–2.06)	0.693	1.45 (0.75–2.77)	0.268
Company location					
	Rural area	1		1	
	Urban area	1.08 (0.71–1.65)	0.705	1.24 (0.79–1.94)	0.348
Desk worker/manual worker					
	Desk worker	1		1	
	Manual worker	0.88 (0.57–1.35)	0.565	0.94 (0.59–1.51)	0.802
Manager/non-manager					
	Non-manager	1		1	
	Manager	0.75 (0.31–1.85)	0.538	0.80 (0.32–2.00)	0.625
Stroke subtype					
	Cerebral infarction	1		1	
	Cerebral hemorrhage	1.28 (0.83–1.97)	0.258	1.13 (0.71–1.78)	0.608
	Subarachnoid hemorrhage	0.67 (0.37–1.19)	0.170	0.54 (0.29–1.02)	0.056
Duration of the first SA					
	< 60 days	1		1	
	> 60 and ≤ 120 days	0.87 (0.53–1.44)	0.599	0.94 (0.56–1.58)	0.824
	> 120 days	1.24 (0.81–1.91)	0.324	1.19 (0.75–1.89)	0.451

As for RSA, during the follow-up period 80 males (31.9%) and 6 females (18.2%) experienced physician-certified RSA. 49 (57.0%) subjects experienced RSA due to recurrent strokes, 18 (20.9%) had RSA due to mental disorders, and 9 (10.5%) had RSA due to fractures (often due to incidents involving steps at a train station or the employee’s house). The cumulative RSA rates at 1, 2, 3, 4, and 5 years were 16.3, 21.0, 25.1, 31.9, and 33.4%, respectively. In total, almost half of the RSA were concentrated within the first year. Two employees experienced RSA due to acute myocardial infarctions (AMI). The other reasons for RSA were as follows: diabetes, gastric cancer, esophageal cancer, epilepsy, inguinal hernia, chronic heart failure, spondylosis, and disc hernia (*n* = 1 in each case). As for the frequency of resignations after RTW, 21 employees resigned. The resignation rates at 1–5 years were 4.9, 7.1, 7.1, 7.6, and 7.6%, respectively. Information on the reasons for resignation could not be obtained in this study.

## Discussion

### Work continuance rate and risk factors for work discontinuation after RTW

As far as we know, there are no other workforce-based Japanese stroke survivor studies after RTW that have examined the frequencies of work continuance, RSA, and resignation, or analyzed the predictors of work discontinuation using survival analysis. This study showed that about 60% of stroke survivors continued to work for 5 years (with the help of RTW support systems) after their RTW. The stroke survivors in this study were supported by the occupational health center, e.g., with a part-time work system, work accommodation, and OP interviews. While there have not been any workforce-based studies examining the work continuance rate after RTW among stroke survivors, we speculate that the work continuance rate of stroke survivors would be lower in smaller companies than the figure reported in this study. Employees working in smaller companies seem to have less ‘protection’ in terms of the capacity of their employer to make ‘reasonable adjustments’ for them to keep working, for example, their ability to conduct OP interviews, ensure a gradual RTW, and transfer the employee to other tasks (Moriguchi et al. 2010). As a future task, we have started to collect data about stroke survivors who returned to work in small- or medium-sized companies, to allow us to compare work continuance rates between companies.

The distribution of work discontinuation due to RSA or resignation observed in this study was similar to the distribution of SA, but Hensing reported that SA data were heavily skewed (Hensing 2004). The Kaplan–Meier curve of the current cohort showed that the work continuance rate declined over time after the employees returned to work. Work discontinuation (i.e., RSA or resignation) was most common in the first year, followed by the second year. The survival curve for RSA among the stroke survivors increased gradually, which was different from the curve for RSA due to depression in the same population (Endo et al. 2013). According to the Kaplan–Meier curve obtained in this study, it might be necessary for occupational health professionals to provide stroke survivors with careful support for 5 years.

Our study showed that older age was significantly associated with the time to work discontinuation (RSA or resignation). The results of our study are consistent with those of previous studies (Perk and Alexanderson 2004; Petty et al. 1998; Saeki et al. 1993; Vyas et al. 2016). For example, older age has been found to be associated with an increased risk of stroke recurrence (Petty et al. 1998), and older stroke survivors might resign more frequently than younger survivors because the period until their retirement is shorter. Vyas et al. reported that older age was associated with a lower likelihood of employment in stroke survivors (Vyas et al. 2016). In studies of RTW rates, older age was demonstrated to be a predictor of a reduced likelihood of returning to work (Perk and Alexanderson 2004; Saeki et al. 1993).

On the other hand, there were no significant associations between work discontinuation and other factors, such as sex, stroke subtype, or the duration of SA. As stroke survivors have various symptoms, which also vary in severity, in future studies we need to use larger samples and have access to clinical and work-related information to allow us to adjust for confounding factors.

### RSA rates and risk factors for RSA among stroke survivors

In this study, the RSA rate at 1 year after the employees’ RTW was 16.3%, which was relatively consistent with the risk of recurrence reported in previous clinical stroke studies (between 7.1 and 21%), while almost half of RSA were caused by recurrent strokes (Mohan et al. 2011). The RSA rate at 5 years was 33.4%, which was consistent with the risk of recurrence in previous studies (between 18.3 and 35.3%) (Mohan et al. 2011). Cabral’s study reported that the overall risk of stroke recurrence was 9%, which was similar to those described in other cohort studies (Cabral et al. 2015).

Two employees experienced RSA due to AMI, which does not seem to be a high number, while previous studies reported that stroke survivors were at higher risk of AMI (Boysen and Truelsen 2000; Talelli and Greenwood 2008).

In this study, 20.9% (18/86) of RSA were caused by mental disorders. Previous studies have reported that mood and emotional disturbances are common symptoms in stroke survivors (Ibeneme et al. 2016; Kim and Choi-Kwon 2000). Mijajlovic reported that strokes worsen cognitive function by inducing post-stroke dementia (Mijajlovic et al. 2017). Balasooriya-Smeekens’s qualitative study demonstrated that most impairments suffered by stroke survivors are “invisible”, e.g., they include fatigue, problems with concentration, memory, and personality changes (Balasooriya-Smeekens et al. 2016).

In this study, 10.5% (9/86) of RSA was caused by fractures, especially fractures caused by incidents involving steps at a train station or the employee’s house. Fracture-induced RSA are more common among stroke survivors than among employees who RTW after depression-induced SA (Endo et al. 2013). Huo reported that bone fractures often occurred in stroke survivors (Huo et al. 2016). Previous stroke survivor studies stated that stroke survivors exhibited lower physical capacities in terms of their ability to walk, use stairs, lift objects, bend, reach, and grasp, and a greater prevalence of activity limitations (Skolarus et al. 2014). We suggest that staggered working hours and home-based teleworking are desirable for reducing the risk of fractures during commuting. It would be better for employers if they ensured that that their workplaces were barrier-free, e.g., if they introduced handrails on both sides of stairways, non-slip floors, and eliminated steps where possible, as this would facilitate the RTW of stroke survivors.

### Strengths, limitations, and implications

First of all, the strengths of the present study included the fact that it was the first large-scale Japanese RTW study of stroke survivors (it involved approximately 300 Japanese employees who returned to work after their first stroke-induced SA). Second, the follow-up rate after RTW was 100%. This meant that this study was not affected by bias associated with loss to follow-up or subject selection, which might have affected other studies. Third, the registered data were based on objective measurements of SA (OP confirmed the clinical validity of the physicians’ certificates and registered any SA using the ICD-10 system). As previous studies of SA were based on self-reported data, the current data would be expected to have greater validity and reliability. As for ICD-10 codes, while many studies were examining ICD-10 codes I60-69 for registering stroke, McCormick et al. reported that ICD codes should not be used, because this broad group of codes, which includes the codes for non-acute and ill-defined cerebrovascular disease, and the late effects of stroke (McCormick et al. 2015). However we registered only “cerebral infarction (I63)”, “cerebral hemorrhage (I61)”, and “subarachnoid hemorrhage (I60)”; based on a physician’s certificate, for stroke as a primary outcome, that might mean our study had better validity. Fourth, the maximum follow-up period was 12.3 years.

However, this study had some limitations that should be taken into account when interpreting the results. The validity of the diagnostic information was one of the major limitations of this study. While OP confirmed the content of the physicians’ certificates, neither clinical data nor imaging findings were used to validate the stroke diagnoses. It remains unclear how we could validate physician-certified causes of RSA during the follow-up period, such as recurrent strokes (57%), fractures (10.5%), or mental disorders (20.9%), especially as the latter are extremely hard for physicians without any psychiatric expertise to diagnose (Davis et al. 2016). In general, the use of administrative data, such as physicians’ certificates as well as ICD-10 information was problematic and this was, moreover, the case in stroke (Tirschwell and Longstreth 2002).

It was not possible for us to use clinical information, e.g., about the stroke site, the impairments caused by the subjects’ strokes, or the severity of the subjects’ disabilities. Harris reported that while the stroke location does not have a strong influence on employment outcomes, functional disabilities, which can change over time, can have a significant impact on them (Harris 2014; Saeki and Hachisuka 2004). In this study, while we assumed that stroke-induced functional disability might result in a longer duration of SA, the multivariate analysis did not detect a significant relationship between the duration of SA and work discontinuation. The work continuance rate among stroke survivors can be affected by clinical factors, while previous clinical studies reported that the recurrence rate was not influenced by clinical parameters (Elneihoum et al. 1998; Kulesh et al. 2011; Mohan et al. 2011). In future studies, the effects of clinical factors on the work continuance rate of stroke survivors after RTW should be evaluated. Second, as the data of this study were mainly collected from large-scale companies, which raises a question about the representativeness of the stroke survivors in the current sample. Employees working in smaller companies seem to have less ‘protection’ in terms of the capacity of their employer to make ‘reasonable adjustments’ to accommodate their RTW (Moriguchi et al. 2010). Thus, caution must be taken when generalizing these results to other populations or other countries. Third, while resignation after RTW was evaluated as work discontinuation in this study, some subjects stopped working for the company being studied, but continued working elsewhere. Thus, it is possible that the index of work discontinuation has been overestimated. Fourthly, the duration of the follow-up period depended on when the stroke-induced sick leave started. This study included stroke survivors that suffered strokes between 2000 and 2011, and the follow-up period ended in December 2012. Thus, not all of the survivors could be followed for more than 1 year. In fact, only the cohort that suffered strokes between 2000 and 2007 could be followed for 5 years or more.

## Conclusion

After RTW, stroke survivors the subjects worked for a mean of 7.0 years. ‘≥50 year group’ were at greater risk of work discontinuation than ‘the ≤ 49 year’.
